# Evaluation of the quality of fixed prosthesis impressions in private laboratories in a sample from Yemen

**DOI:** 10.1186/s12903-020-01294-1

**Published:** 2020-11-04

**Authors:** Nusaiba M. Al-Odinee, Mohsen Al-Hamzi, Ibrahim Z. Al-shami, Ahmed Madfa, Abdulwahab I. Al-Kholani, Yazeed M. Al-Olofi

**Affiliations:** 1 Department of Conservative Dentistry, Faculty of Dentistry, Sana’a University, Sana’a, Yemen; 2 Department of Conservative Dentistry, Faculty of Dentistry, Thamar University, Dhamar, Yemen; 3 Restorative and Prosthodontic Department, College of Dentistry, University of Science and Technology, Sana’a, Yemen; 4 Department of Restorative Dental Science, College of Dentistry, University of Ha’il, Ha’il, Kingdom of Saudi Arabia

**Keywords:** Impressions, Fixed prosthesis, Laboratories, Quality, Yemen

## Abstract

**Background:**

Fixed prosthodontics require an accurate impression for the teeth and the area to be restored for the laboratory to fabricate the desired restoration without mistakes. This study evaluated the quality of impressions received by private laboratories for the fabrication of fixed prosthesis by describing the frequency of clinically detectable errors and by analyzing association between the various factors involved.

**Methods:**

165 impressions were collected from four dental laboratories. Jaw involved, type of tray, size of tray, number of prepared units, type of impression materials, techniques and viscosity in case of elastomeric impressions and type of prosthesis requested were recorded. Data referring to errors and visible defects including errors in finish line, in preparation area, in silicone impression technique and blood in impression were also documented. Factors affecting errors present were also assessed. Association between dentist gender and experience years and impression errors was assessed. Chi square and Fisher exact tests used to examine the association between categorical variables and outcomes.

**Results:**

The total of error considering not immediately pouring as an error. Alginate was the most impression used. of impressions evaluated (50.9%), 97% were have at least one visible error; 92.1% had errors in finish line, 53.9% had errors in preparation area and (72.8%) of elastomeric impressions were have at least one error in technique. Blood in impression was detected in 52.1% of impressions. Significant association was found between material type and errors in finishing line and preparation area. Significant relationships were found between gender and errors in silicone impression technique (p < 0.05).

**Conclusion:**

Within the limitations of this study, high frequency of detectable errors was found in fixed prosthesis impressions received by private dental laboratories. This high frequency is of serious concern, as this will result in poor fitted fixed prosthesis provided to patients.

## Background

Well-adapted crowns are required for the durability of prepared teeth. There is a 3% risk for endodontic and caries failure at a single abutment tooth for the crown, but several prepared teeth for fixed bridges are at a 15% risk for endodontic and caries failure [1]. The transfer of perfect impressions to the dental laboratories is always done by dentists as part of the daily routine of prosthesis construction in fixed prosthodontics [2, 3]. The lab technicians work on fabrication of prosthesis on casts obtained from the already-taken impressions, which are taken by various techniques, to make them ready for the first trial on patients. As mentioned above, the techniques used for fabrication fixed prosthesis impressions are numerous including the following: (1) the copper band technique, (2) the single viscosity technique (monophase technique), (3) the single-step technique or dual viscosity technique (in which impression materials of 2 viscosities are applied at the same time) or (4) the two-step technique (using material of dissimilar viscosity in each step, in which the impression is recorded in two steps) [4–8].

Considering the extent of the impression, dental impressions are classified to complete or full dental impressions and segmental or partial dental impressions. A wide diversity of trays are used for recording dental impression: prefabricated or stock trays (total and partial), custom-made trays and dual arch trays (total and partial) with several impression materials such as condensation cured silicone, addition-cured silicone or polyether [9].

Obtaining an ideal soft-tissue displacement and a perfect impression for a fixed dental prosthesis is one of the most difficult procedures until know in dentistry [2, 3]. Since impressions reproduce both the gingiva and the teeth, success is based on complete knowledge of the anatomy of the periodontal tissues, making an accurate preparation (predominantly at the finish line), using the appropriate impression material and proper technique [7, 10, 11].

The results from several studies exhibit enhancement in properties and accuracy of contemporary impression materials [12, 13]. However, the quality of dental impressions received by dental laboratories for the manufacturing of fixed dental prosthesis distinctly has remained inadequate [14–20]. In 1997, results of the evaluation of 290 impressions from 4 commerical dental laboratories presented that 36% of the examined impressions had detectable errors [15]. After two years, the results of another survey presented that the quality of 50% of impressions and dies sent to dental laboratories are inadequate [21]. In 2005, an evaluation of 193 dental impressions sent to eleven dental laboratories results in 89% of evaluated impressions had at least 1 visible error [14]. In 2014, results of an evaluation of 200 impressions sent to four dental laboratories in Malaysia resulted in 64.5% of examined impressions to be inadequate or unacceptable [16]. A recent survey in North Carolina, USA showed that 86% of the evaluated impressions had at least 1 visible error, and 55% of the detectable errors were critical errors affecting the preparation finish line [17].

Although variation exists between impression materials, all necessitate perfect technique in gingival displacement, correct placement of the impression material around the prepared teeth, and proper use of impression trays [3]. One of the main causes of unacceptable impressions is poor soft tissue displacement [14, 15, 22]. Another of the main causes of an unacceptable fixed prosthesis is a deficiency of knowledge of the principles of impression recording and knowledge of what constitutes an accurate impression [23]. Correct handling of the impression material is possibly more important than properties of the material itself in detecting the final accuracy of the impressions [12, 13, 24].

On the basis of personal communication with technicians, many technicians claim they are noticing a decline in the quality of impressions they have been receiving over the years [17]. Therefore, it is important to examine whether dentists critically examine impressions before transferring them to the laboratories. In addition, no study in Yemen has yet evaluated the quality of fixed prosthesis impressions. Therefore, this study aimed to determine the quality of fixed prosthesis impressions collected from four private laboratories in Sana’a city, Yemen.

## Methods

This study was an observational cross-sectional study evaluating the quality of impressions received by private laboratories for the fabrication of fixed prosthesis in Sana’a city, Yemen. The evaluation and examination of an impressions were performed from August 2019 to October 2019.

First of all, calibration was performed by the examiner (N. M.) with the supervisor (Associate Prof. in Fixed Prosthodontics M. A. with 29 experience years) by initially evaluating and inspecting eleven impressions rejected from Faculty of Dentistry, Sanaa University- Yemen as being unacceptable for fixed prosthesis fabrication. Then reliability was performed by evaluating the other nine impressions by the examiner and supervisor separately and testing agreement using the kappa test. Kappa test result in the overall agreement value was 0.9. After that, discussion has been performed with the supervisor about existing 0.1 differences so that all decisions in the errors of impression were standardized.

Four laboratories were selected according to geographic distribution, one in the north of Sana’a, another in the south of Sana’a and two laboratories in central Sana’a. In the previously mentioned geographical locations, well known laboratories that receive a large number of impressions and deals with largest number of dentists have been chosen. In each laboratory, all fixed prosthesis impressions received were evaluated without selection or rejection. The impression was examined by one examiner. A total of 165 impressions were collected and examined. Impressions without specific requests for FPDs and those that have been poured with stone before examination were excluded from examination.

Sample size determination was based on using this equation [n = z^2^ p (100—p)/e^2^] to calculate sample size; the minimum sample size was 149. With confidence level at 95% (1.96), acceptable sample error (5) and frequency of errors in samet’s study (89.1%) [14].

An impression evaluation form modified from previous studies was used to collect the data [14–17, 25]. All impressions were tested immediately before manipulation by dental technicians. When multiple abutments were recorded in one impression, error on any abutment was recorded as a defect for the whole impression. The assessment of the impressions was performed using 3.5 × magnification loupes under room lightening without the aid of additional illumination.

For each impression, jaw involved, type of tray, size of tray, number of prepared units, type of impression materials, techniques and viscosity in case of elastomeric impressions and type of prosthesis requested were evaluated. In order to differentiate between condensation and addition silicone, the products of condensation silicone or addition silicone present in Sana’a city are confined and known for their distinctive colors and thus it was easy to distinguish between them.

Errors and noticeable defects were also documented, including errors in finish line area that were considered the critical errors including voids, bubbles, tears in the finish line, tissue over the finish line, pull or fold were recorded or if the finish line is accurate it was scored as no error. Most of the finish lines were knife-edge and difficult to detect in impressions, in this case, the end of prepared tooth was considered the finish line. Mistakes in the preparation area were also documented as voids, bubbles, tray show through preparation or poor details. Other errors in tray and material including blood on impression also were reported. Faults of impression technique were also evaluated as inadequate mixing, stepped impression, heavy-bodied or putty material exposure through the wash material, inadequate union of material, lack of wash materials in finish line area or lack of polymerization. Retention of impression materials to the tray was recorded as adequate or inadequate. The gingival retraction was also reported as adequate or inadequate. The gingival retraction was considered adequate if the full finish line was visible with the light body was penetrated into the gingival sulcus.

Information about dentists who took the impressions was taken from technicians. This information helps to relate errors present to the dentist’s gender and years of experience. There was no attempt to know the dentist name. This information was kept strictly confidential and used for the purpose of scientific research only. The dentists’ years of experience were categorized to two categories (≤ 10 years) and (> 10 years) and the dentist gender was categorized to male and female. Working location either private or government clinic was also recorded.

The analysis of the data was performed using SPSS 21.0 for Windows (SPSS Inc., Chicago, IL, USA). Descriptive analysis and frequency tables were used to present data. A chi-square and Fisher exact tests were conducted to examine the association between categorical variables and outcomes.

## Results

The total of error considering not immediately pouring as an error. Of the 165 impressions evaluated, 105 (63.6%) were maxillary impressions and 60 (36.4%) were mandibular impressions. Plastic stock trays were 152 (92.1%) and the metal stock tray was 13 (7.9%). Full arch trays were 164 (99.4%). The distribution of impression materials was 84 (50.9%) alginate, 47 (28.5%) condensation silicone and 34 (20.6%) addition silicone. Of the 165 impressions, 65 (39.4%) contained four or more prepared abutments, 45 (27.3%) contained two prepared abutments, 38 (23%) contained 1 abutment and 17 (10.3%) contained three abutments.

165 impressions are included in the study from 165 dentists, 112 (67.9%) was male, 53 (32%) was female, proportion of male to female was 2.11: 1.

According to experience years, 128 (77.6%) had ≤ 10 years and 37 (22.4%) had more than 10-year experience.

Most of the impressions evaluated (N = 125) were mainly from two dental laboratories because of the abundance of impressions in these laboratories and lack of impressions in the other two laboratories.

**Fig. 1:**
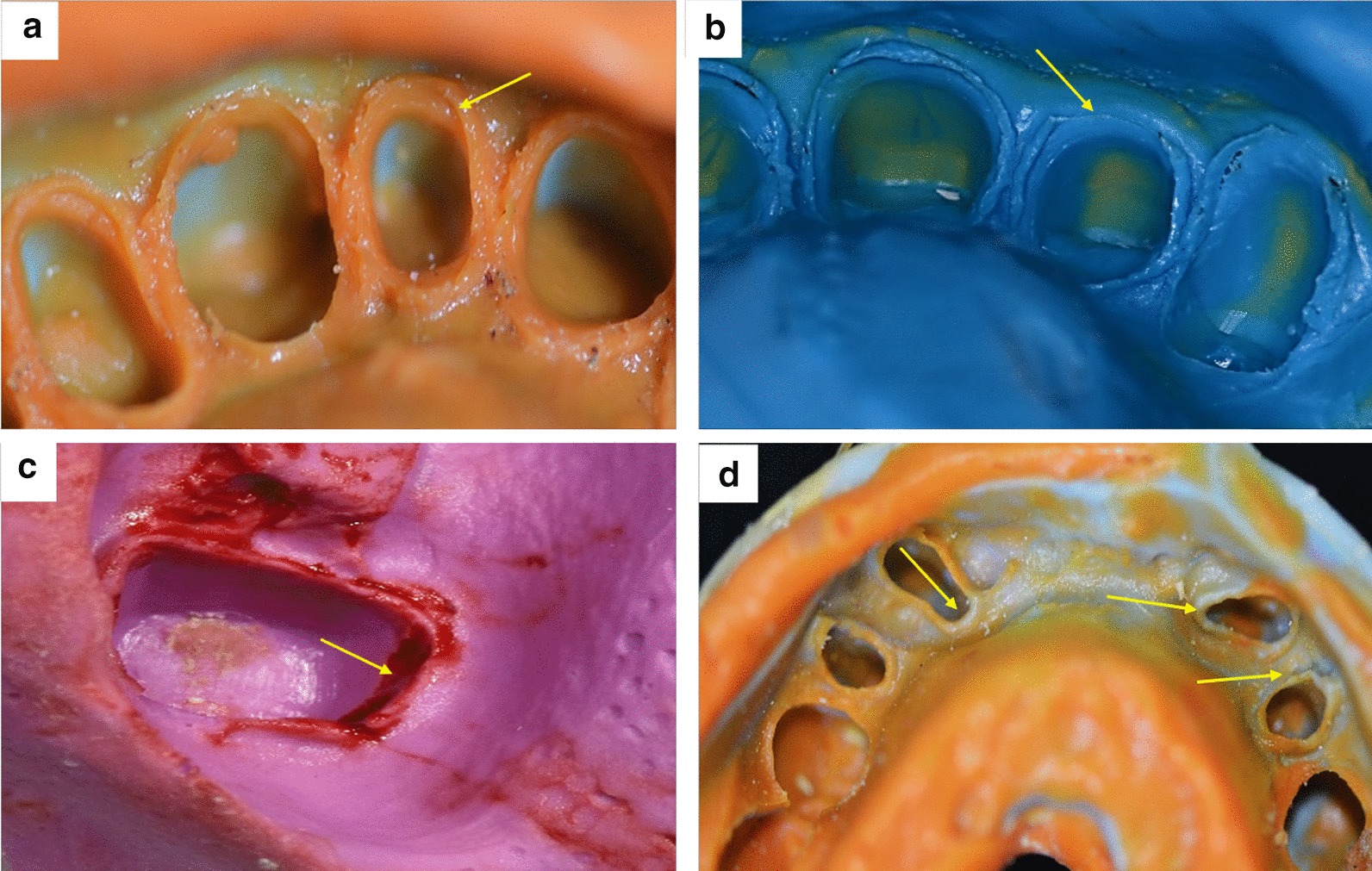
Examples of the errors that found in study sample; **a** Finish line errors; tissue over finish line, **b** Finish line errors; tears in finish line, **c** Errors in preparation area; voids, **d** Errors in silicone impression technique; lack of wash materials in finish line

The total error of impressions was (97%) considering not immediately pouring as an error. The most common error in finish line was bubbles (69.1%), followed by voids (43.6%). In general, (92.1%) of impressions had errors in the finish line as exhibited in Table 1 and Fig. 1. Of the impressions evaluated, (53.9%) had errors in the preparation area. Bubble (34.5%) were the most common errors (Table 1). There was a significant association in finish line errors and errors in the preparation area in alginate’s compared with silicone impressions (p < 0.05) as represented in Table 2.

**Table 1:** Errors of impressions

	F	%
*Type of error in the finish line*		
Voids in finish line	72	43.6
Bubbles in finish line	114	69.1
Tear in finish line	29	17.6
Tissue over finish line	19	11.5
Pull or fold in finish line	54	32.7
Inadequate gingival retraction	147	89.1
*Type of error in the preparation area*		
Voids in preparation area	45	28.3
Bubbles in preparation area	54	34.0
Tray show through preparation area	21	13.2
Poor details	149	6.3
Inadequate retention of material to tray	8	4.8
*Blood in impression*	86	52.1

**Table 2:** Association between the type of errors and types of impression material

	Impression material						p-value
	Alginate		Addition silicone		Condensation silicone		
	F	%	F	%	F	%	
*Voids in finish line*							
Yes	59	81.9	3	4.2	10	13.9	0.000
No	25	26.9	31	33.3	37	39.8	
*Bubbles in finish line*							
Yes	70	61.4	13	11.4	31	27.2	0.000
No	14	27.5	21	41.2	16	31.4	
*Tear in finish line*							
Yes	16	55.2	5	17.2	8	27.6	0.848
No	68	50.0	29	21.3	39	28.7	
*Tissue over finish line*							
Yes	6	31.6	5	26.3	8	42.1	0.191
No	78	53.4	29	19.9	39	26.7	
*Pull or fold in finish line*							
Yes	22	40.7	9	16.7	23	42.6	0.020
No	62	55.9	25	22.5	24	21.6	
*Gingival retraction*							
Adequate	2	14.3	11	78.6	1	7.1	0.000
Inadequate	82	55.8	20	13.6	45	30.6	
*Supragingival finish line*	0	0.0	3	75.0	1	25.0	
*Total impressions with errors in the finish line*							
Yes	84	55.3	24	15.8	44	28.9	0.000
No	0	0.0	10	76.9	3	23.1	
*Retention of material to tray*							
Adequate	78	49.7	34	21.7	45	28.7	0.308
Inadequate	6	75.0	0	0.0	2	25.0	
*Voids in preparation area*							
Yes	31	67.4	4	8.7	11	23.9	0.016
No	53	44.5	30	25.2	36	30.3	
*Bubbles in preparation area*							
Yes	35	61.4	7	12.3	15	26.3	0.084
No	49	45.4	27	25.0	32	29.6	
*Tray show through preparation area*							
Yes	20	95.2	0	0.0	1	4.8	0.000
No	64	44.4	34	23.6	46	31.9	
*Poor detailed impressions*							
Yes	5	50.0	1	10.0	4	40.0	0.633
No	79	51.0	33	21.3	43	27.7	
*Total impressions with errors in the preparation area*							
Yes	58	65.2	9	10.1	22	24.7	0.000
No	26	34.2	25	32.9	25	32.9	
*Blood in impression*							
Yes	69	80.2	11	12.8	6	7.0	0.000
No	15	19.0	23	29.1	41	51.9	

Of 81 elastomeric impression materials, 65 (80.2%) was a double step and 16 (19.8%) was a single-step technique. All of the elastomeric impression 81 was putty wash technique. Of 81 elastomeric impression materials, errors in impressions technique were 59 (72.8%). The most common error was heavy-bodied materials exposure through wash material (69.1%) as shown in Table 3.

**Table 3:** Errors of silicone impression technique

Type of errors of silicone impression technique	F	%
Total impressions with errors	59	72.8
Inadequate impression materials mixing	22	27.2
Stepped impression	4	4.9
Lack of wash materials in finish line area	13	16
Heavy bodied materials exposure through wash material	56	69.1
Inadequate union of materials	0	0.0
Lack of polymerization	0	0.0

Considering errors in the silicone impression technique, there was a significant association of errors among females compared to males (*p* < 0.05). There was an increased percentage of errors in silicone impression technique among dentists with experience years ≤ 10 years with a significant difference (*p* < 0.05). Lack of wash materials in finish line area and Heavy bodied materials exposure through wash material were more in dentist with experience years > 10 years with significance difference too (*p* < 0.05) as shown in Additional file 1: Table S1.

This study presented that there was non-significant association between errors in the finishing line and in the preparation area and dentist’s gender or years of experience (p > 0.05) as shown in Additional files 2, 3: Tables S2 and S3.

## Discussion

This study was the first in Yemen to evaluate the quality of fixed prosthesis impressions and to determine the factors that affect errors present in impressions. It is very important for dentists to self-evaluate the impressions after taking them as a fundamental step for the clinical success of fixed prosthesis. In this study, the impressions were evaluated according to these criteria: errors in finish line errors, retention of material to tray, errors in the preparation area, gingival retraction, errors in silicone impression technique, blood in impression and retention of material to the tray.

This study showed that 97% of impressions received by the dental laboratories had at least one detectable error which is in agreement with previous studies [14–17].

Marginal detail is the most critical aspect of the impression. Failure to record the appropriate details of the finish line of the preparation will result in incorrect prosthetic fit. The errors in the finish line were considered critical errors. In this study 152 out of 165 (92.1%) impressions were had at least one visible error in the finish line. This finding was higher than Rau's study in which the finish line area had at least one visible error in 55% of the evaluated impressions [17].

According to the present study, (69.1%) of impressions had bubbles in the finish line. This percentage was in agreement with Samet’s study (40.4%) [14]. Bubbles occurs as a result of air entrapment during mixing of material, tray-loading, syringing or tray placement and can negatively affect the fit of the prosthetics [24, 26]. Voids in this study were detected in (43.6%) of impressions evaluated. Voids usually larger and less sharp in definition and occurs due to fluid accumulation [24]. This result was consistent with Samet’s study (50.7%) in Israel and more than Rau’s study in North Carolina, USA (24.8%) [14, 17].

Pull or fold in the finish line was detected in (32.7%) of impressions evaluated. It is often produced at the gingival aspect when impression material pasts its working time (no longer in its most fluid state) or when the impression material fails to adapt to the teeth [24]. Tear in the finish line was presented in (17.6%) of impressions (Fig. 1b). Marginal tears can result when a syringing material with inadequate tear strength is used, using a light body PVS in a thin deep sulcus, or the impression is removed prior to the complete setting of the syringing material [24]. The tissue over the finish line was detected in (11.5%) of impressions evaluated (Fig. 1a). This result was less than Rau’s study (49.09%) [17]. That is due to the difficulty of detecting this error because most of the finish line types in evaluated impressions were knife-edge and it is difficult to detect in impression to determine if the tissue is covering the finish line.

Accurate impressions of the margins can only be anticipated with appropriate gingival displacement, margin design, margin placement, and moisture control. In tooth-supported fixed prosthesis, impression making requires an accurate record of the prepared finish line area, especially in cases where the preparation margin is located at the same level of gingiva or sub-gingiva [27–29]. In this study, the adequate gingival retraction was adequate only in (8.5%) of impression evaluated.

The result of this study presented that errors in the preparation area were detected in (53.9%) of impression evaluated. The most common error in the preparation area was bubbles (34.5%) followed by voids (27.9%) as shown in Fig. 1c. Air bubbles are resulted during mixing, while voids are resulted due to moisture or debris on the oral tissues [30]. This percentage was in agreement with Rau's study [17] in which voids percentage was (13.3%). Voids may be large enough to affect the long-term success of the luting cement, which must now fill a wider space. The prosthetic material may also be thinner than recommended. This can be more critical when using all-ceramic materials, as they require minimum thicknesses to perform as expected [24]

The contaminated impression is considered a principle possible route of spread of infection from the patient in the clinic to dental technicians [31]. Disinfection of impression materials should be mandatory to prevent cross-infection. The impression should be rinsed with water and then disinfected [32]. The present study found that blood was in (52.1%) of impressions evaluated. This finding was in the same line with a study in Sudan in which blood was clear in (68.9%) [33] and higher than that in North Carolina, USA in which (14.70%) of impression was soiled with blood [17].

The results of this study indicated that the most generally used impression materials by the general practitioners for their crown and bridgeworks were alginate impression materials. These practices weren't concurrent with the practice worldwide where the most commonly used materials by the general practitioners for their crown and bridgeworks were elastomeric impression material [14, 16, 17, 20]. Alginate is not accurate enough for fixed partial dentures but used for partial framework impressions or provisional restorations [34, 35]. In addition, usually the alginate impressions were sent to the laboratory covered by wet paper; this method is not considered ideal because impression can easily absorb water from the wet paper and consequently deform before pouring [12].

PVSs are the impression materials of choice for fixed prosthodontics. However, in this study Addition silicone only account for (20.6%). The widespread use of alginate may be related to their cheap price, lack of knowledge about the proper use of silicone impression materials and/or to the dentist’s lack of knowledge about their limitations.

This study was reported that all of the silicone impressions were recorded using the putty wash technique. The most commonly used impression techniques for putty wash are one-step and two-step techniques. The result of this study showed that out of 81 silicone impressions, (80.2%) were two-step techniques and (19.8%) were the one-step technique. Hung et al. [36] and Idris et al. [6] concluded that impression accuracy is technique independent and the differences between techniques were not considered to be clinically important. On the other hand, other studies stated that the impression technique is a significant factor in determining the accuracy of the impression [37–40]. The one-step putty-wash technique requires less chair-side time. The two-step putty-wash technique produces some more precise castings [41]. The two-step putty-wash technique has been reported to be more accurate than the one-step putty-wash technique [37–39, 41] because there is uniform space for the light-body material to polymerize and the details are captured by the light-body material only [37, 40, 41].

The most common error in the silicone impression technique was heavy-bodied materials exposure through wash material in (69.1%) of impressions evaluated, and this finding was in agreement with Samet's study (44%) [14]. This error in double-step technique may indicate that there is no uniform space for the light body either before putty impression taking or during cutting space for the light body after putty impression taking. In the single-step technique, this error due to the putty tends to push the light-body wash away from the prepared tooth [42].

Lack of wash materials in the finish line area represent the third most common errors in silicone impression technique (7.9%) (Fig. 1D), this finding was in agreement with Rau’s study in North Carolina, USA (16%) [17]. The light body materials are able to record fine detail of 25 µm or less [43], but the putty materials, in general, cannot record fine detail at the 25-µm level and are required only to reproduce the detail of 75 µm [42].

In order to increase the accuracy of final impressions, the dimensional stability of an impression tray is also a contributing factor. Trays should have good stability along a period of time and do not portray any permanent deformation between impression taking and pouring stage [8]. The use of a soft plastic stock tray cannot be considered an ''observable defect,’’ although there is a likelihood for inaccuracies due to the flexible nature of these trays [15, 44]. The result of this study presented that (92.1%) of impression was recorded using a plastic tray as found in previous studies [14, 17]. The widespread use of such trays may be related to their cheap cost and/or to the dentist’s lack of knowledge about their limitations.

In general, most of the impressions evaluated were had at least one error. There are many reasons for the high incidence of unacceptable impressions sent to the laboratories. It could either be clinicians' factors, material properties factors or patient factors. As for clinicians, it is either due to the lack of knowledge and experience, poor manipulation of the impression material, lack of attention to details, low awareness on the need for critical self-evaluation [16], early removal of the impressions prior to complete setting which means that it is possible that impressions are often removed from the mouth when the dentist ‘‘feels’’ that the material has polymerized, ignoring the polymerization time recommended by the manufacturer [14]. Or even financial constraint could be the possible underlying reasons for these unacceptable impressions being sent to the laboratory [16].

Dentists involved in this study are graduated from different Yemeni dental schools which have varying crown and bridge prerequisites, and requirements for graduation. In general, teaching of crown and bridge is started from the third year as “fixed prosthodontics (I)” in which a preclinical annual course is taught. In this course, the dental students are required to perform different type of preparations “all metal, metal-ceramic, all ceramic and partial veneer crown “on artificial acrylic teeth, also they taught to perform the one step and two step putty wash technique by using condensation silicone.

In the following fourth year, “fixed prosthodontics (II)” is taught, which is a clinical annual course in which students have to perform many simple single crown cases. Finally, on the fifth year of the dental program, clinical fixed prosthodontics is taught as “fixed prosthodontics (III)” in which students are required to perform three or four unite bridges, custom made post and fiber post. The students are required to record final impression by using condensation silicone and due to the high cost of addition silicone, many dental schools do not provide this material for students.

Teaching the fixed prosthodontics is considered highly qualified but unfortunately many dentists do not keen to update their information after graduation. Also, the absence of control by the medical council may contribute to these unacceptable impressions.

There is a tendency for errors of silicone impression technique to be more in female dentists than males. This finding may be explained by that the number of male dentists is more than females, and thus their error percentage decreased. This result may need more studies to prove or disprove it.

There was an increased percentage of errors occurred in silicone impression technique among dentists with less than 10 years of experience with a significant difference. Lack of wash materials in finish line area and Heavy bodied materials exposure through wash material were more in dentists with more than 10 years of experience years with a significant difference too. This can be explained by that experience years is not a factor that influences the quality of impression or a larger number of samples may be necessary to study this association.

This study presented that there was non-significant association between errors in the FL and in the preparation area and dentist’s gender or years of experience. A larger number of samples may be necessary to study this association.

## Limitation

In this study, four dental laboratories were chosen according to geographic distribution. But, most of the impressions evaluated were mainly from two dental laboratories because of the abundance of impressions in these laboratories and lack of impressions in the other two laboratories.

This study aimed to detect the association between working location (private or governmental) and impressions errors. But all impressions evaluated were from private dental clinics and this factor was not evaluated. Further studies are needed to detect the association between working location (private or governmental) and impressions errors.

Also, this study evaluated only the outcome of impression recording, but more studies are required to examine the quality of the dies and the outcome of the definitive restoration.

Information about dentists who took impression was taken from technicians, so there was a possibility of potential bias.

## Conclusions

Within the limitations of this study, the high frequency of detectable errors was found in fixed prosthesis impressions received by dental laboratories. This high frequency is of serious concern, as this will result in poorly fitted fixed prosthesis provided to patients. A more critical evaluation of impressions by the dentists themselves is recommended. This can be improved by attending short lectures or intense courses to update and improve their knowledge and skills on current techniques in fixed prosthodontics. These intense courses may be organized by respected organizations for the general practitioners in the future.

## Supplementary information

**Additional file 1.** **Table S1.** Association between gender and experience years of the dentist and the errors in silicone impression technique.

**Additional file 2.** Association between gender and experience years of the dentist and the type of error in the finish line.

**Additional file 3.** Association between gender and experience years of the dentist and the type of error in the preparation area.

## Data Availability

The data used and/or analysed during the present study is included as supplementary file. Any additional data and material are available from the corresponding author on reasonable request.

## Abbreviations

FPD: Fixed partial denture
PFM: Porcelain fused to metal
PVS: Polyvinylsiloxane
FL: Finish line
