# Supplementary material for: Evaluation of the quality of fixed prosthesis impressions in private laboratories in a sample from Yemen
Source: BMC Oral Health. 2020 Nov 4;20:304. doi: 10.1186/s12903-020-01294-1 (PMC7640478; doi:10.1186/s12903-020-01294-1)
Supplement: Supplementary file 1 — Additional file 1. Table S1. Association between gender and experience years of the dentist and the errors in silicone impression technique. [file 12903_2020_1294_MOESM1_ESM.docx]

**Table S1.** Association between gender and experience years of the dentist and the errors in silicone impression technique.

|  | | Gender | | | |  |
| --- | --- | --- | --- | --- | --- | --- |
|  |  | Male | | Female | | P- value |
|  |  | F | % | F | % |  |
| Total impressions with errors silicone impression technique | Yes | 34 | 75.6% | 25 | 69.4% | .001 |
|  | No | 11 | 24.4% | 11 | 30.6% |  |
| Inadequate impression materials mixing | Yes | 7 | 15.6% | 15 | 41.7% | .003 |
|  | No | 38 | 84.4% | 21 | 58.3% |  |
| Stepped impression | Yes | 2 | 4.4% | 2 | 5.6% | .000 |
|  | No | 43 | 95.6% | 34 | 94.4% |  |
| Lack of wash materials in finish line area | Yes | 8 | 17.8% | 5 | 13.5% | .002 |
|  | No | 37 | 82.2% | 32 | 86.5% |  |
| Heavy bodied materials exposure through wash material | Yes | 33 | 73.3% | 23 | 63.9% | .002 |
|  | No | 12 | 26.7% | 13 | 36.1% |  |
|  | | Years of experience | | | |  |
|  |  | ≤ 10yrs | | More than 10 | | P- value |
|  |  | F | % | F | % |  |
| Total impressions with errors silicone impression technique | Yes | 46 | 67.6% | 13 | 100% | .031 |
|  | No | 22 | 32.4% | 0 | 0.0% |  |
| Inadequate impression materials mixing | Yes | 18 | 26.5% | 4 | 30.8% | .210 |
|  | No | 50 | 73.5% | 9 | 69.2% |  |
| Stepped impression | Yes | 4 | 5.9% | 0 | 0.0% | .170 |
|  | No | 64 | 94.1% | 13 | 100% |  |
| Lack of wash materials in finish line area | Yes | 9 | 13.0% | 4 | 30.8% | .046 |
|  | No | 60 | 87.0% | 9 | 69.2% |  |
| Heavy bodied materials exposure through wash material | Yes | 43 | 63.2% | 13 | 100% | .017 |
|  | No | 25 | 36.8% | 0 | 0.0% |  |
