# Supplementary material for: Evaluation of the quality of fixed prosthesis impressions in private laboratories in a sample from Yemen
Source: BMC Oral Health. 2020 Nov 4;20:304. doi: 10.1186/s12903-020-01294-1 (PMC7640478; doi:10.1186/s12903-020-01294-1)
Supplement: Supplementary file 2 — Additional file 2. Association between gender and experience years of the dentist and the type of error in the finish line. [file 12903_2020_1294_MOESM2_ESM.docx]

**Table S2.** Association between gender and experience years of the dentist and the type of error in the finishing line.

|  | | Gender | | | | |
| --- | --- | --- | --- | --- | --- | --- |
|  |  | Male | | Female | | P – value |
|  |  | F | % | F | % |  |
| Errors in finish line | Yes | 102 | 91.1% | 50 | 94.3% | .438 |
|  | No | 10 | 8.9% | 3 | 5.7% |  |
| Voids in finish line | Yes | 50 | 44.6% | 22 | 41.5% | .709 |
|  | No | 62 | 55.4% | 31 | 58.5% |  |
| Bubbles in finish line | Yes | 73 | 65.2% | 41 | 77.4% | .075 |
|  | No | 39 | 34.8% | 12 | 22.6% |  |
| Tear in finish line | Yes | 20 | 17.9% | 9 | 17.0% | .894 |
|  | No | 92 | 82.1% | 44 | 83.0% |  |
| Tissue over finish line | Yes | 13 | 11.6% | 6 | 11.3% | .961 |
|  | No | 99 | 88.4% | 47 | 88.7% |  |
| Pull or fold in finish line | Yes | 39 | 34.8% | 15 | 28.3% | .471 |
|  | No | 73 | 65.2% | 38 | 71.7% |  |
| Retention of material to tray | Adequate | 108 | 96.4% | 49 | 92.5% | .270 |
|  | Inadequate | 4 | 3.6% | 4 | 7.5% |  |

|  | | Years of experience | | | | | |
| --- | --- | --- | --- | --- | --- | --- | --- |
|  |  | ≤ 10 years | | More than 10 years | | P – value | |
|  |  | F | % | F | % |  |  |
| Errors in finish line | Yes | 115 | 89.8% | 37 | 100.0% | .200 | |
|  | No | 13 | 10.2% | 0 | 0.0% |  |  |
| Voids in finish line | Yes | 56 | 43.8% | 16 | 43.2% | .776 | |
|  | No | 72 | 56.2% | 21 | 56.8% |  |  |
| Bubbles in finish line | Yes | 87 | 68.0% | 27 | 73.0% | .771 | |
|  | No | 41 | 32.0% | 10 | 27.0% |  |  |
| Tear in finish line | Yes | 22 | 17.2% | 7 | 18.9% | .903 | |
|  | No | 106 | 82.8% | 30 | 81.1% |  |  |
| Tissue over finish line | Yes | 13 | 10.2% | 6 | 16.2% | .389 | |
|  | No | 115 | 89.8% | 31 | 83.8% |  |  |
| Pull or fold in finish line | Yes | 42 | 32.8% | 12 | 32.4% | .894 | |
|  | No | 86 | 67.2% | 25 | 67.6% |  |  |
| Retention of material to tray | Adequate | 123 | 96.1% | 34 | 91.9% | .390 | |
|  | Inadequate | 5 | 3.9% | 3 | 8.1% |  |  |
| Pearson Chi-Square test. | | | | | | |  |
