# Supplementary material for: Evaluation of the quality of fixed prosthesis impressions in private laboratories in a sample from Yemen
Source: BMC Oral Health. 2020 Nov 4;20:304. doi: 10.1186/s12903-020-01294-1 (PMC7640478; doi:10.1186/s12903-020-01294-1)
Supplement: Supplementary file 3 — Additional file 3. Association between gender and experience years of the dentist and the type of error in the preparation area. [file 12903_2020_1294_MOESM3_ESM.docx]

**Table S3.** Association between gender and experience years of the dentist and the type of error in the preparation area.

|  | | Gender | | | | |
| --- | --- | --- | --- | --- | --- | --- |
|  |  | Male | | Female | | P – value |
|  |  | F | % | F | % |  |
| Errors in preparation area | Yes | 61 | 56.5% | 25 | 49.0% | .378 |
|  | No | 47 | 43.5% | 26 | 51.0% |  |
| Voids in preparation area | Yes | 30 | 27.8% | 15 | 29.4% | .831 |
|  | No | 78 | 72.2% | 36 | 70.6% |  |
| Bubbles in preparation area | Yes | 38 | 35.2% | 16 | 31.4% | .636 |
|  | No | 70 | 64.8% | 35 | 68.6% |  |
| Tray show through preparation area | Yes | 16 | 14.8% | 5 | 9.8% | .384 |
|  | No | 92 | 85.2% | 46 | 90.2% |  |
| Poor details | Yes | 8 | 7.4% | 2 | 3.9% | .503 |
|  | No | 100 | 92.6% | 49 | 96.1% |  |

|  | | Years of experience | | | | |
| --- | --- | --- | --- | --- | --- | --- |
|  |  | ≤ 10 years | | More than 10 years | | P – value |
|  |  | F | % | F | % |  |
| Errors in preparation area | Yes | 62 | 55.4% | 27 | 50.9% | .060 |
|  | No | 50 | 44.6% | 26 | 49.1% |  |
| Voids in preparation area | Yes | 30 | 26.8% | 16 | 30.2% | .524 |
|  | No | 82 | 73.2% | 37 | 69.8% |  |
| Bubbles in preparation area | Yes | 39 | 34.8% | 18 | 34.0% | .863 |
|  | No | 73 | 65.2% | 35 | 66.0% |  |
| Tray show through preparation area | Yes | 16 | 14.3% | 5 | 9.4% | 1.000 |
|  | No | 96 | 85.7% | 48 | 90.6% |  |
| Poor details | Yes | 8 | 7.1% | 2 | 3.8% | .054 |
|  | No | 104 | 92.9% | 51 | 96.2% |  |
| Pearson Chi-Square test. | | | | | | |
